# Inertial Tail Effects during Righting of Squirrels in Unexpected Falls: From Behavior to Robotics

**DOI:** 10.1093/icb/icab023

**Published:** 2021-04-30

**Authors:** Toshihiko Fukushima, Robert Siddall, Fabian Schwab, Séverine L D Toussaint, Greg Byrnes, John A Nyakatura, Ardian Jusufi

**Affiliations:** 1 Locomotion in Biorobotic and Somatic Systems Group, Max Planck Institute for Intelligent Systems, Heisenbergstraße 3, Stuttgart 70569, Germany; 2 Institute of Biology, Humboldt University of Berlin, Philippstrasse 13, Haus 2, 10115 Berlin, Germany; 3 Department of Biology, Siena College, 515 Loudon Road, Loudonville, NY 12211, USA

## Abstract

Arboreal mammals navigate a highly three dimensional and discontinuous habitat. Among arboreal mammals, squirrels demonstrate impressive agility. In a recent “viral” YouTube video, unsuspecting squirrels were mechanically catapulted off of a track, inducing an initially uncontrolled rotation of the body. Interestingly, they skillfully stabilized themselves using tail motion, which ultimately allowed the squirrels to land successfully. Here we analyze the mechanism by which the squirrels recover from large body angular rates. We analyzed from the video that squirrels first use their tail to help stabilizing their head to visually fix a landing site. Then the tail starts to rotate to help stabilizing the body, preparing themselves for landing. To analyze further the mechanism of this tail use during mid-air, we built a multibody squirrel model and showed the righting strategy based on body inertia moment changes and active angular momentum transfer between axes. To validate the hypothesized strategy, we made a squirrel-like robot and demonstrated a fall-stabilizing experiment. Our results demonstrate that a squirrel’s long tail, despite comprising just 3% of body mass, can inertially stabilize a rapidly rotating body. This research contributes to better understanding the importance of long tails for righting mechanisms in animals living in complex environments such as trees.

## Introduction

Arboreal mammals navigate a highly three dimensional and discontinuous environment. Many arboreal mammals including primates and some squirrels spend much time feeding and moving on narrow, flexible terminal branches (e.g., Orkin and Pontzer 2011; Nyakatura 2019). Tail use in the arboreal context has been observed in several species (e.g., McClearn 1992; Cunha and Vieira 2002; Jusufi et al. 2008, [Bibr icab023-B13][Bibr icab023-B35]) and despite specializations, fractures from falls have been documented, for example, in primates (Jurmain 1997; Nakai 2003). Squirrels are widely observed to be among the most maneuverable arboreal animals. Moreover, there are inherent risks in utilizing the terminal branch niche. Narrow and flexible branches could break underfoot and the bounding locomotion of squirrels on narrow branches (Young and Chadwell 2020) could make recovery to a stable substrate more difficult, resulting in falls from height. Further, unlike many other arboreal mammals, rodents and squirrels have limited ability to grasp the substrate (Nyakatura 2019; Toussaint et al. 2020), potentially increasing the risk of falling and making the ability to reorient while airborne paramount to alighting safely. In a recent YouTube video which went “viral,” squirrels (*Sciurus carolinensis*) voluntarily visiting a YouTuber’s garden cross a parkour to earn a food reward (Rober 2020). When the squirrels attempted to reach a feeder, they were suddenly catapulted off the track. Interestingly, the animals did not appear to be harmed by the catapulting event, and quickly returned to the parkour to try again to reach the food reward. We did not intend to experimentally reproduce this for ethical reasons though, this catapulting event yielded intriguing observations regarding the self-righting behavior of an arboreal specialist, the common gray squirrel. We observed from the video that the body rotations of the animals were initially uncontrolled, but they skillfully rotated their tails and stabilize their bodies, finally landed on safe places with stable postures.

To date, aerial righting reflexes have been described in various animals such as cats (Marey 1894; Kane and Scher 1969), rats (Laouris et al. 1990), lizards (Jusufi et al. 2010; Jusufi et al. 2008), and stick insects (Jusufi et al. 2011). Interestingly, many animals could use their tail to support righting behavior. By actively moving their tail, which has a given mass and shape, they effectively use its rotational inertia to produce counter-acting forces allowing them to perform body righting. It has been shown some lizards are able to use tail inertia to control roll rotation during free-fall (Jusufi et al. 2008, 2010) and pitch orientation during leaping (Libby et al. 2012). Also, wild cheetahs have been hypothesized to use their tail inertia to assist reorientation of their bodies when they rapidly change directions while pursuing prey (Wilson et al. 2013). Interestingly, in arboreal primates, which exhibit various tail morphologies, the active use of tail inertia for self-righting seems to be intrinsically related with the relative length of the tail and locomotor mechanisms such as grasping or jumping ability (Dunbar 1988; Young et al. 2015).

Meanwhile, robotic righting using a tail has also been explored. Inertial tails have been used for robot steering (Kohut et al. 2012; Pullin et al. 2012; Patel and Braae 2013) and tail interaction with terrain has enabled robots to steer (Casarez and Fearing 2018) and recover from a rolled-over posture (Casarez and Fearing 2017). In robotic aerial righting, tail inertia has been used to control roll orientation during free-fall (Jusufi et al. 2010) and pitch orientation during jumping (Johnson et al. 2012; Libby et al. 2012; Yim and Fearing 2018). Additionally, yaw orientation has also been controlled by an inertial tail, in concert with aerodynamics (Kohut et al. 2013).

In these studies, the aerial righting reflex has been observed recovering from an upside-down orientation during a free-fall, modification of leaping orientation, or body steering during a free-fall. In these instances, rotations were limited to one plane and righting schemes were from (semi-)static to static in rotational kinetics; initial conditions were states without or with only minimal angular momentum and ended the righting process in a similarly static state, after a period in motion. On the other hand, in the squirrels’ righting maneuvers described here, they were initially subjected to large unexpected angular velocities (more than 1080°/s). In spite of this, they successfully stabilized their bodies and landed with their feet in desired orientation by moving their bodies and tails three-dimensionally in the air and spinning the tails multiple times in less than 1 s. Therefore, these righting maneuvers started from a dynamic state and resulted in a static body state.

In this paper, we address the righting mechanism of squirrels following this particular falling event, from its behavioral analysis to the conception of a model-based robot enables to reproduce this complex aerial righting motion pattern. First, we extract tail and body motions from the video (available on a social media platform in Rober 2020) and model the squirrel’s righting *in silico*. Then, we numerically analyze their righting maneuver and perform experimental validation with a squirrel-like robot.

## Behavioral analysis

**Fig. 1:**
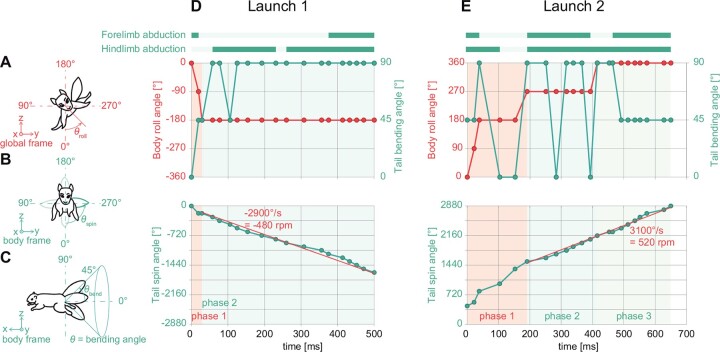
Behavioral analysis results. **A–C**) Definitions of body roll angle, tail spin angle, and tail bending angle, respectively. **D**) and **E**) Body and tail motions in launch 1 and launch 2. The mean tail spinning speeds were estimated by slope of the approximated red lines via least squares method.

To analyze the righting maneuvers of the catapulted squirrels, we extracted their aerial motions, especially focusing on their tail movements. The squirrels were catapulted off the track two times, which we will refer as launch 1 (at 17:51 in Rober 2020) and launch 2 (at 16:19 in Rober 2020). In the video, the launch events are shown from a single point of view, with the camera positioned relatively laterally regarding the launching site. However, as this raw dataset was collected under non-controlled conditions, we could not obtain the exact position nor the distance of the camera from the squirrels. Also, the launches events are both shown in the video with a regular speed of 24 fps, and in slow motion. We extracted and analyzed all the sequences of interest using the Adobe Premiere Pro CS6 software, and extracted the speed of slow motion at 183 fps. To ensure that this data were analyzed as rigorously as possible despite its limitation, we collected the timestamps at which particular angular positions occurred (e.g., 0°, 90°, 180°, 270°, and 360°) along with the direction of the rotation, for three variables of interest: the angular movement of the body in the terrestrial referential frame (global frame), defined as the body roll angle (Fig. 1A); the angular movement of the tail in the squirrel’s referential frame, defined as the tail spin angle (Fig. 1B); and the bending angle of the tail relative to the longitudinal axis of the body, defined as the tail bending angle (Fig. 1C). Analysis of launches both demonstrate a succession of defined phases, which are based on head, body, and tail rotating movements relative to each other, as follows.

### Launch 1

Duration of the flight was 0.50 s (Fig. 1D). The individual left the launcher with large roll angular momentum. The body and limbs were steady from a defined point of the launch, and the tail exhibited clear rotatory movements in relation to the body frame. At the time of launch, the body was positioned in pronation regarding the global frame (ventral side facing the ground). Here, based on the body roll angle $\theta_{\text{roll}}$, the righting pattern was divided in two phases.

#### Phase 1: rotating head and body

Duration of the phase was 29 ms. The individual exhibited a negative directional body rotation in relation with the *y*–*z* plane in the global frame (Fig. 1A, D), so that the body ended up in a supine posture (dorsal side facing the ground). The tail didn’t rotate actively.

#### Phase 2: stabilized head and body, rotating tail

Duration of the phase was 471 ms. The body stayed quite steady ($\theta_{\text{roll}}$ was constant), with the head facing the landing site. The head and body were aligned in supination regarding the ground, but with a notable general folding of the body. The hindlimbs moved actively, with a succession of abduction and adduction, whereas the forelimbs stay folded (adducted) toward the chest. The tail exhibited high amplitude of rotatory movements in the same direction as the initial body rotation in the body frame (Fig. 1B, D) and made complete rotations in *y*–*z* plane in the body frame. The tail was also bent between 45° and 90° (Fig. 1C, D). Four distinct complete rotations of the tail were counted during this phase.

### Launch 2

Launch 2 was overall more complicated, involving simultaneous rotations and movements of the body, limbs, and tail (Fig. 1E). The individual was launched with large initial roll angular momentum, as well as slight pitch angular momentum. Duration of the flight was 0.67 sec. At the time of the launch, the body was again positioned in pronation regarding the global frame (ventral side facing the ground). Here, based on the head movement in the global frame and the body roll angle $\theta_{\text{roll}}$, the righting pattern was divided in three phases.

#### Phase 1: rotating head and body

Duration of the phase was 169 ms. There were complex movements directly following the catapulting of the individual. Body and head rotated in positive direction with respect to the global frame (Fig. 1A, E) and the tail also rotated in positive direction relative to the squirrel body frame (Fig. 1B, E). Forelimbs and hindlimbs exhibited successive abduction and adduction. The body ended up perpendicular to the ground.

#### Phase 2: stabilized head, rotating body and tail

Duration of the phase was 284 ms. The head was locked down toward the landing site. The body still rotated in positive direction (Fig. 1A, E), and maintained a global pronated position (ventral face of head and body facing the ground). Forelimbs and hindlimbs still exhibited succession of abduction–adduction. The tail actively rotated in positive direction (Fig. 1B, E) with a bending position which varied during the phase (Fig. 1C, E).

#### Phase 3: stabilized head and body, rotating tail

Duration of the phase was 213 ms. Both head and body were locked toward the landing site ($\theta_{\text{roll}}$ was constant), in a pronated posture (ventral side facing the ground). Forelimbs and hindlimbs stayed abducted until landing. The tail actively rotated in positive direction, with high amplitude of movement, and with complete rotations in relation to the body plane (Fig. 1B, E). The tail was bent between 45° and 90° (Fig. 1C, E). Four distinct complete rotations of the tail were counted during combining phases 2 and 3.

In summary, the squirrels first coordinately controlled their bodies and tails to recover from the uncontrolled initial rotation and stabilize the head and visually fix the landing site. Then, they continuously spun the tails, inducing a counter moment, to slow down the body rotation and eventually stop the body rotation to prepare themselves for landing. Interestingly, in both launches the tails stayed relatively straight and rotated in a plane which is orthogonal to the animal’s main axis (*y*–*z* plane in the body frame in Fig. 1A–C).

## Model analysis

### Modelization

Using kinematic information extracted from the preliminary observations, we used a model to predict squirrel kinematics on unexpected ballistic trajectories.

**Fig. 2:**
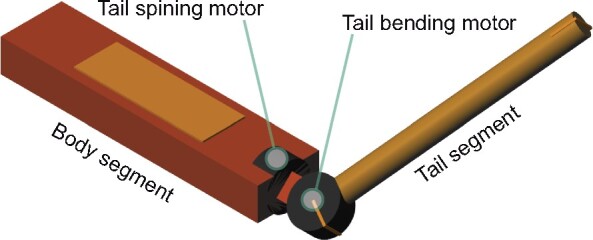
The multi body model for the simulation analysis.

We quantified the mass and dimensions of body segments of a specimen of *Sciurus carolinensis* (Table 1). Moments of inertia of different appendages have been estimated by treating each segment as a uniform rod (Moment of inertia = Mass×Length^2^/12). We also referred each appendage to its base (the point at which the appendage attaches to the body) with the parallel axis theorem by assuming the limb is fully extended (i.e., all segments parallel). This represents the maximum possible moment of inertia of the limb. Examining at the values, the tail has significantly greater inertia than the four limbs, and has an inertia moment at its base which is 25% of torso moment of inertia. While the hind limbs have large inertia, they have a significantly smaller range of motion, so the calculated moments of inertia about the appendage base are not representative of their utility as inertial controls. We then made a three dimensional multibody model of the animal using Simscape Multibody in MATLAB/Simulink (Fig. 2). To compute the tail inertia effect alone, the model consists simply of a solid body and a solid tail segment, both with uniform density. The body segment is approximated by a cuboid and the tail segment by a cylinder. The model has two rotational degrees of freedom (DOF), which were for the tail bending and the tail spin, driven by motor torque. The first DOF is attached to the body, such that the tail bending motor is attached to the output of the tail spinning motor. Dimensions, mass, and inertia of the model were set based on the measured anatomy (Table 2).

**Table 1:** Dimensions and mass of each segment of the body of *Sciurus carolinensis*, with estimated moments of inertia about the appendage center of gravity and about the base of the appendage (where the appendage meets the body, e.g., a shoulder). To segment the body, vertebral spines were palpated and the body was divided anterior to the spines on thoracic vertebrae 4 and 13.

Segment	Part	Length	Width	Mass	Appendage moment of	Moment of inertia
					inertia about own CoM	about appendage base
		(mm)	(mm)	(g)	(10^–6^ kg m^2^)	(10^–6 ^kg m^2^)
Whole animal		–	–	341		
Tail		215	–	11	34.5	99.9
	T1 (anterior)	72	–	6.1		
	T2 (mid)	71.5	–	3.1		
	T3 (posterior)	71.5	–	1.6		
Head		72	38	27	4.4	17.5
Body		164	56	235	444.9	n/a
	B1 (anterior)	54	48	67		
	B2 (mid)	55	56	106		
	B3 (posterior)	55	53	62		
Left forelimb		–	–	8.4	0.9	4.0
	LF arm	49	–	3.8		
	LF forearm	52	–	3.4		
	LF hand	35	–	1.2		
Right forelimb		–	–	9.9	1.2	6.3
	RF arm	49	–	5.3		
	RF forearm	54	–	3.4		
	RF hand	36	–	1.2		
Left hindlimb		–	–	25	8.0	61.6
	LH thigh	50	–	16		
	LH leg	66	–	5.7		
	LH foot	62	–	3.2		
Right hindlimb		–	–	24.3	7.3	55.3
	RH thigh	49	–	15.6		
	RH leg	66	–	5.5		
	RH foot	56	–	3.2		

**Table 2:** Specifications and variable definitions of the multi body model. Each moment of inertia with respect to its CoM.

Name	Variable	Axis	Value
Body mass (kg)	$m_{b}$	–	0.330
Tail mass (kg)	$m_{t}$	–	0.011
Body dimensions (m)	$l_{\text{bl}},l_{\text{bw}},l_{\text{bh}}$	l, w, h	0.236 × 0.056 × 0.030
Tail dimensions (m)	$l_{\text{tl}},l_{\text{tw}}$	l, w (diameter)	0.215 × 0.020
Body (inc. head) moment	$I_{\text{br}}$	Roll	$1.11\times 10^{-4}$
of inertia about body	$I_{\text{bp}}$	Pitch	$1.55\times 10^{-3}$
CoM ($\text{kg}\cdot m^{2}$)	$I_{\text{by}}$	Yaw	$1.62\times 10^{-3}$
Tail moment of inertia	$I_{\text{tl}}$	Longitudinal	$4.26\times 10^{-5}$
about tail CoM ($\text{kg}\cdot m^{2}$)	$I_{\text{ts}}$	Cross-sectioned	$5.50\times 10^{-7}$

### Inertial stabilization

To analyze the inertial effect on body stabilization, which was observed in phase 2 of the launch 1 and phases 2 and 3 of the launch 2, we spun the simulated tail at speed with 90° bending angle. The model was initially given −360°/s of roll angular velocity in the global frame to emulate the conditions in launch 1. In this study, the model was in free-fall, and aerodynamic effects were ignored to see the inertial effect in isolation.

**Fig. 3:**
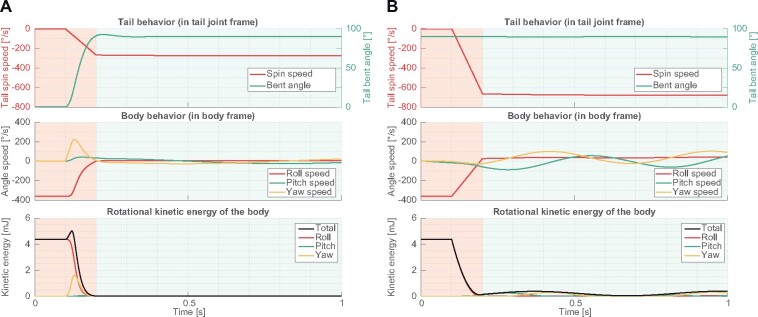
Roll axis stabilization using tail rotational inertia. **A**) Starting from tail straightened posture ($\theta_{\text{bend}}=0^{{}^{\circ}}$). **B**) Starting from tail bent posture ($\theta_{\text{bend}}=90^{{}^{\circ}}$).

As expected, after the model started to spin the tail (at *t *=* *0.1 s), body roll rotation quickly stopped (Fig. 3A). Body initial angular momentum was transferred to tail spin angular momentum, stabilizing the body rotation. In this test, the tail was initially straightened (tail bending angle, $\theta_{\text{bend}}=0^{{}^{\circ}}$). As the model bent the tail outward due to centrifugal inertia, a body movement in the yaw axis was observed. However, due to the large difference in moment of inertia between the body $I_{\text{by}}$ and tail $I_{\text{tl}}$, the body yaw rotation was small.

In contrast, when the model initially bent the tail at $\theta_{\text{bend}}=90^{{}^{\circ}}$, the required angular velocity of the tail to stabilize the body was larger than the test starting from $\theta_{\text{bend}}=0^{{}^{\circ}}$ (Fig. 3B). This was explained by conservation of angular momentum during the righting. Initial angular momentum of the body and the tail needed to be transferred to tail angular momentum. In the case where $\theta_{\text{bend}}=0^{{}^{\circ}}$, the equation of conservation of angular momentum can be represented as follows:

(1) $$I_{\text{br}}\omega_{\text{init}}+I_{\text{ts}}\omega_{\text{init}}=I\prime_{\text{tl}}\omega_{\text{req}\_0}.$$

(2) $$\omega_{\text{req}\_0}=\left(\frac{I_{\text{ts}}}{I\prime_{\text{tl}}}+\frac{I_{\text{br}}}{I\prime_{\text{tl}}}\right)\omega_{\text{init}}.$$

Here, $I_{\text{br}},I_{\text{ts}},I_{\text{tl}}^{`},\omega_{\text{init}},\text{and }\omega_{\mathrm{req\_}0}$ respectively stand for body roll moment of inertia, tail longitudinal moment of inertia around body roll rotational axis, initial angular velocity, and required tail spinning speed. On the other hand, the required tail spinning speed $\omega_{\text{req}\_90}$ in the simulation of $\theta_{\text{bend}}=90^{{}^{\circ}}$ can be represented as follows:

(3) $$I_{\text{br}}\omega_{\text{init}}+I\prime_{\text{tl}}\omega_{\text{init}}=I\prime_{\text{tl}}\omega_{\text{req}\_90}.$$

(4) $$\omega_{\text{req}\_90}=\left(1+\frac{I_{\text{br}}}{I\prime_{\text{tl}}}\right)\omega_{\text{init}}.$$

Since $I_{\text{ts}}/I\prime_{\text{tl}}<1$, the required tail spinning speed to stabilize the body gets significantly larger in the simulation of $\theta_{\text{bend}}=90^{{}^{\circ}}$ than in $\theta_{\text{bend}}=0^{{}^{\circ}}$. In the video, it was also observed that the squirrels straightened their tails, just before they were launched in both of the launches 1 and 2, even though they were bending their tails before the launches. The squirrel may potentially adopt this pose to reduce the angular momentum of the tail imparted at launch. Additionally, undulation in pitch and yaw axes in Fig. 3(B) was caused by unbalanced body and tail inertia alignment (see the Supplementary Video).

Furthermore, we explored the effect of starting from the straightened tail in a point of body and tail relationship. Here, the tail longitudinal moment of inertia around body roll rotational axis ($I\prime_{\text{tl}}$) can be represented by a combination of the tail longitudinal moment of inertia around its center of mass (CoM) $I_{\text{tl}}$ and moment of inertia of a mass point around the body roll rotational axis $m_{t}{\left(\frac{1}{2}l_{\text{tl}}\right)}^{2}$.

(5) $$I\prime_{\text{tl}}=I_{\text{tl}}+m_{t}{\left(\frac{1}{2}l_{\text{tl}}\right)}^{2}.$$

Using Equations (2), (4), and (5) and following equations representing each moment of inertia, speed ratio of the required tail spin is described as below

(6) $$I_{\text{br}}=\frac{m_{b}}{12}(l_{\text{bw}}^{2}+l_{\text{bh}}^{2}).$$

(7) $$I_{\text{tl}}=\frac{m_{t}}{4}\left(\frac{l_{\text{tw}}^{2}}{4}+\frac{l_{\text{tl}}^{2}}{3}\right).$$

(8) $$I_{\text{ts}}=\frac{m_{t}}{8}l_{\text{tw}}^{2}.$$

(9) $$\text{Then},\;\;\;\frac{\omega_{\text{req}\_0}}{\omega_{\text{init}}}=\sigma +\frac{6l_{\text{tw}}^{2}}{16l_{\text{tl}}^{2}+3l_{\text{tw}}^{2}}.$$

(10) $$\frac{\omega_{\text{req}\_90}}{\omega_{\text{init}}}=\sigma +1.$$

(11) $$\text{where}\;\;\;\sigma =\frac{4m_{b}\left(l_{\text{bh}}^{2}+l_{\text{bw}}^{2}\right)}{m_{t}\left(16l_{\text{tl}}^{2}+3l_{\text{tw}}^{2}\right)}.$$

**Fig. 4:**
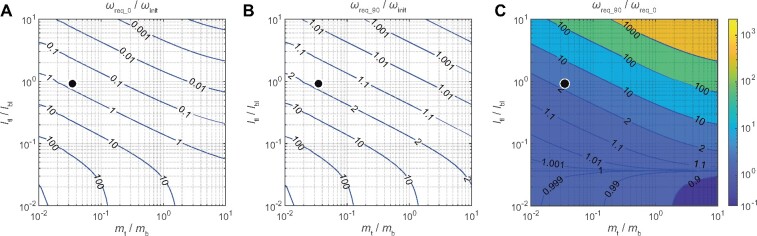
Initially straightened tail effect. Figures show speed ratios of required tail spin for initial rotation to stop body rotation with varying tail mass $m_{t}$ and length $l_{\text{tl}}$. The tail mass axis and the tail length axis are normalized by body mass $m_{b}$ and body length $l_{\text{bl}}$. Black points show tail–body proportion of the studied model. **A**) With the model initially straightening the tail. **B**) With the model initially bending the tail at 90°. **C**) Gain comparison of the straightened tail and bent tail. In the area of $\omega_{\text{req}\_90}/\omega_{\text{req}\_0}<1$, tail moment of inertia along the length direction is smaller than the one along width direction.

Next, to explore gain sensitivity of the effect in tail characterization, we varied tail mass $m_{t}$ and tail length $l_{\text{tl}}$ and plotted contour maps of $\omega_{\text{req}\_0}$ and $\omega_{\text{req}\_90}$ with normalized tail mass and length (Fig. 4A, B). The maps showed when the tail mass and length were large, $\omega_{\text{req}\_0}/\omega_{\text{init}}$ converged to 0, and $\omega_{\text{req}\_90}/\omega_{\text{init}}$ converged to 1. Fig. 4C showed gain difference of $\omega_{\text{req}\_90}/\omega_{\text{init}}$ and $\omega_{\text{req}\_0}/\omega_{\text{init}}$. When tail length was long relative to body length, the straight tail effect worked effectively, even though the tail mass was very small relatively to the body mass (Fig. 4C). For instance, the studied model’s tail proportion was marked as black points in the maps. Although the tail mass was 3.3% of the body mass, due to 91% length of the tail relative to the body, the required tail spin speed could be decreased by 60% by the effect ($\omega_{\text{req}\_0}/\omega_{\text{init}}=0.66,\omega_{\text{req}\_90}/\omega_{\text{init}}=1.65$). This result would support the hypothesis that the squirrels straightened their tails to reduce the angular momentum imparted at the moment of launching. As a note, although the initial bent tail posture would decrease the initial body rotation speed, since the catapult was continuously driven by external power supply (air compressor), therefore the initial rotation speed cannot be simply defined by conservation of angular momentum and affected by position on the catapult, feet slipping, weight balance, reflex of muscle activity, and so on. Thus, we assumed same initial rotation speed nevertheless tail bending angle $\theta_{\text{bend}}$.

### Angular momentum capacitor

**Fig. 5:**
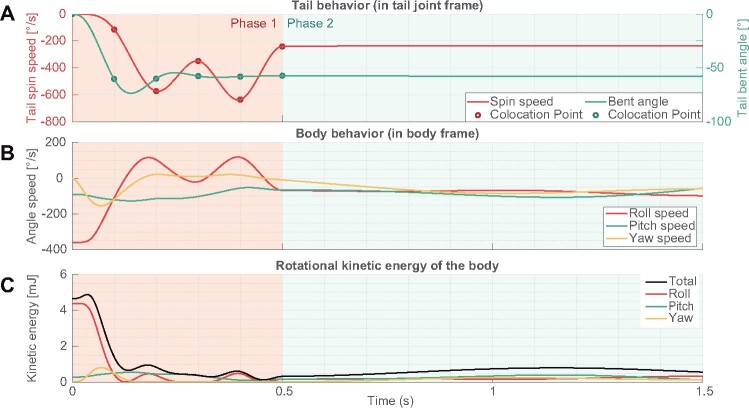
Three dimensional stabilization. The model was initially given −360°/s roll and −90°/s angular velocity. **A**) Optimized tail trajectories in the spin and bent actuation points. **B**) Body behavior of the model. **C**) Rotational kinetic energy of the body.

To analyze the complex transient period of the phase 1 in the launch 2, we set an initial condition which emulates the kinematics of launch 2 and explore tail actuation to stabilize the body. We gave the model initial pitch angular velocity, in addition to the initial roll angular velocity, specifically −360°/s of roll and −90°/s of pitch angular velocity. Here, to seek tail actuation patterns which stabilize the body, we used a genetic algorithm (GA) to optimize the tail trajectory. In this optimization to bound the dimensionality of the problem passed to the GA, collocation points were used to define a trajectory (Kelly 2017) in tail bending angle and tail spinning angular rate of the actuators. These collocation points enabled us to describe continuous time series data by some discrete inputs (Fig. 5A). In this optimization, the collocation points were set at time *t_i_* in simulation time *t* and each collocation point was interpolated via a cubic spline curve. *t_i_* was set as $t_{i}=\left\{0.1,0.2,0.3,0.4,0.5\right\}$s in the simulation time. After *t *>* *0.5, the target value was held at the value at *t*_5_ to emulate the phase 2. An array of values of the collocation points was passed to the GA as a genetic representation to optimize the tail trajectory. The cost function to be minimized by the GA was defined as mean of the total rotational kinetic energy of the body during 0.5<t≤1.5 (the black line in Fig. 5C).

In the output trajectory generated by the GA, the body reduced 87% of kinetic energy with the optimized tail actuation (initially 4.65 mJ). Especially, body roll rotation was stably stopped after the actuation of the tail (Fig. 5B). As with the simulation of the launch 1, first the body roll rotation speed was reduced by rapidly bending the spinning tail and yaw rotation speed temporally increased. Then, after roll angular velocity again increased (though much smaller relative to the initial roll angular velocity), the body was stabilized when the model continuously rotated the tail. Although rapid tail bending increased the body’s yaw axis angular velocity, bending tail at the same time increased the moment of inertia in body roll axis, thereby resisting body roll rotation. In the squirrels’ cases, since the body’s moment of inertia in yaw axis $I_{\text{by}}=1.62\times 10^{-3}$ is much larger than tail longitudinal inertia moment $I_{\text{tl}}=4.26\times 10^{-5}$, the strategy that they employ (bending the tail rapidly to increase rotational inertia) was effective. Observations in natural habitats also found that the kangaroo rat continuously spin their tail during unexpected jump (Schwaner et al. 2021). These observations highlighted that during the jump, when the flexible tail was shortened in the rotational plane by bending like an arc, the tail’s counter inertia effect on the body was decreased.

**Fig. 6:**
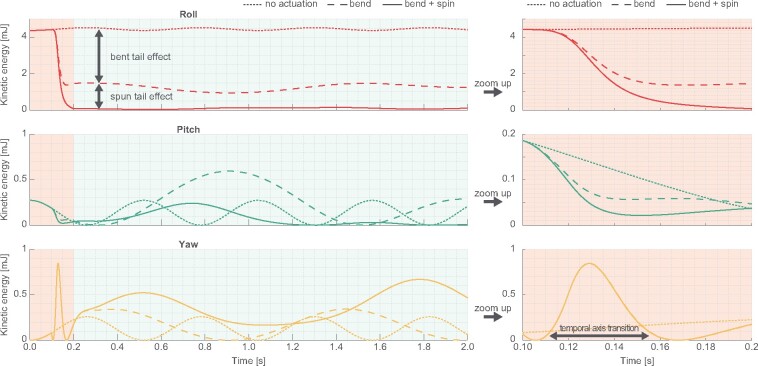
Effect of tail bending and spinning on body stabilization. The model was initially given −360${}^{{}^{\circ}}$/s roll and −90°/s pitch angular velocity. The plots showed the body kinetic energy in each rotational axis. The right hand plots expandingly show the left hand plots during dynamic transition phase of the actuation for 0.1 < time < 0.2. In no actuation’ the model stayed in the initial posture. In ‘bend’ the model started bending the tail to $\theta_{\text{bend}}=90^{{}^{\circ}}$ at time = 0.1 s and completed the actuation at time = 0.2s. In bend + spin’ the tail started being bent and spun at time = 0.1 s and completed the actuation at time = 0.2 s (same profile as Fig. 3A).

To analyze this effect in simplified form, we compared three types of tail actuation patterns (Fig. 6). With tail bending (‘bend’), increased roll inertia ($I_{\text{br}}\to I_{\text{br}}+I\prime_{\text{tl}}$) slowed down the body roll rotation. In the meantime, yaw angular velocity temporary increased, but the amount of increased yaw velocity was smaller than reduced roll velocity because of large moment of inertia $I_{\text{by}}$. Also pitch rotation speed was slightly reduced. This may be caused by three dimensional coordination of each rotational axis. When the tail was bent and spun together, roll rotation speed was reduced further. The difference between bend’ and ‘bend + spin’ showed the amount of the tail counter inertia effect. Although the roll and pitch angular velocities were decreased additionally during the transition period, the amount of increased yaw angular velocity was the same as for the “bend” condition. From these results it is supposed that, using the large yaw axis moment of inertia of the body, the squirrels temporally transit rotational axis from roll and pitch to yaw and rapidly bend the tails to earn larger moment of inertia as total system, which enables them to slow down the rotation speed and to gaze at the landing points. Thus, large yaw moment of inertia of the body works like a capacitor for angular momentum.

## Robot analysis

To further substantiate this model and analyze the underlying mechanics of the self-righting behavior, we developed an abstracted squirrel-like robot complete with an actuated tail to replicate self-righting behavior.

### Robot design

**Fig. 7:**
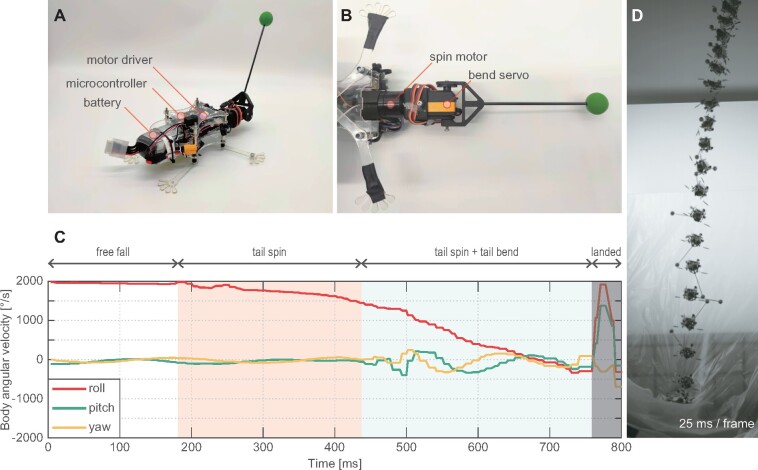
A) Overview of the squirrel-like robot. **B**) Tail actuation mechanism. **C**) Tail inertial stabilization. The spinning bent tail stabilized the body rotation before its landing. **D**) A sequential photo of the dropping robot. After the tail started to be spun, body rotation speed decreased.

The robot was designed based on the model used in the “Model analysis” section (Fig. 7A). Proportion of body and tail in mass and length was set as same as the measured squirrel (92% body length and 3.4% body weight, Table 3). The robot consisted of a body segment and a tail segment, which were connected by 2 × 1 DOF servo joints for tail bending and tail spinning (Fig. 7B). The spinning joint was actuated by brushless motors (GB2208, T-MOTOR) with a motor driver (Basic 30A ESC, BlueRobotics) and the bending joint was actuated by a digital servo (SC-1251MG, SAVÖX). Servo control and data recording to an SD card were done with an ARM Cortex-M7 microcontroller board (Teensy 4.1, PJRC). The robot has an inertial measurement unit (BNO055, Bosch) on the body. Power was supplied from a 22.2 V lithium polymer battery (TATTU 1050 mAh 22.2 V, Gens Ace) for the spinning motor control, and with an L7805 linear voltage regulator IC for the bending servo control and the microcontroller. The tail segment was composed of a carbon fiber pole and a counter weight, such that moment of inertia of the tail could be adjusted by changing pole length and weight mass. The robot could also adjust body moment of inertia by applying some ballast on the ends of limb parts. The robot body was constructed from laser cut polyethylene sheets, with brackets and adapters for the motors and electronics prototyped with a Markforged 3D printer.

**Table 3:** Specifications of the squirrel-like robot

Name	direction	value
Body mass (g)	–	415
Tail mass (g)	–	14
Body dimension (mm)	l, w, h	190, 70, 60 (excl. feet parts)
Tail dimension (mm)	l, w	175, 25 (at the counter mass ball)

### Experimental setup

The robot was dropped from 3.5 m height with initial roll angular velocity. The tail was initially set as straight ($\theta_{\text{bend}}=0^{{}^{\circ}}$) and after a free fall period, the robot started to rotate tail and bend the tail in feed forward control. Tail target bending angle was $\theta_{\text{bend}}=90^{{}^{\circ}}$ and target spinning speed was at a constant speed. After the righting measurement, the robot straightened the tail again to protect the servos from impact damages. Robot's behaviors were recorded by a high-speed camera (S-Motion 2987, AOS) at 500 fps and the IMU on the robot’s body at 250 Hz.

### Results

The robot achieved aerial righting behaviors using its inertial tail spinning, which were similar to that observed in the squirrels and predicted by the simulation studies (Fig. 7C, D, also see the Supplementary Video). First the robot free-fell with constant body roll angular velocity, then after the robot started to spin the tail, the body roll angular velocity also started to decrease. Tail bending additionally decreased the body angular velocity with slight increasing of body yaw and pitch angular momentum, as with the phenomenon observed in the simulation study. In the end, the body angular velocity was converged when the tail spinning speed got constant.

## Discussion

### Righting mechanism with an inertial tail

Based on the combined results from the behavior, simulation, and robot analysis, we found that the squirrels used the following strategy to stabilize their body:

#### Phase 0: straightened *tail to reduce inertia*

At the moment when they are catapulted, they straighten their tails to reduce the initially given moment of inertia (Fig. 4).

#### Phase 1: rapid tail bending to gain larger inertia

**Fig. 8:**
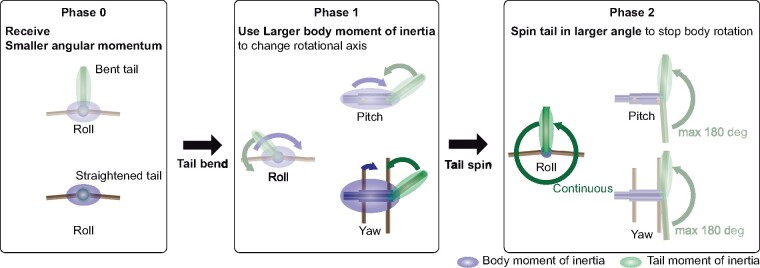
Squirrels’ righting strategy. Phase 0: At the moment when they are catapulted, they straighten their tails to reduce the initially given moment of inertia. The two figures represent schematic views of a squirrel from posterior side. Phase 1: Using large body inertia in the yaw axis as an angular momentum capacitor, while they transfer rotational axis from roll and pitch to yaw, they rapidly bend the tail to obtain larger moment of inertia in roll axis in total. Phase 2: They spin the bent tail to reduce their body angular momentum with transferring it from the body to the tail. Roll rotational axis allows them to spin their tails continuously instead of being mechanically limited in pitch and yaw rotation.

Using large body inertia on yaw axis as an angular momentum capacitor, while they transfer rotational axis from roll and pitch to yaw, they rapidly bend the tails to obtain larger moment of inertia in roll axis in total (Fig. 8). This allows the body to rotate more slowly and enables them to gaze at the landing points (Fig. 6).

#### Phase 2: counter inertia stabilization

They spin their bent tails to reduce their body angular momentum while transferring it from the body to the tail (Fig. 3). Roll rotational axis allows them to spin their tails continuously instead of being mechanically limited in pitch and yaw rotation (Fig. 8).

The initial phase (phase 0) consisting in tail straightening has an important role for phases 1 and 2 successful achievement. It allows to change rotational axis by the rapid bending of the tail in phase 1 (Figs. 3A and 5) and also maximizes the effect of counter inertia of tail spinning in phase 2 (Fig. 4C). As an additional advantage to transit rotational axis to yaw, they can also control the amount of moment of inertia independently from tail actuation by limb adduction and abduction as has been shown during the locomotion of mammals and birds (Kilbourne 2014; Kilbourne and Carrier 2016).

### Aerodynamic effect

In the literature, the righting mechanism was mainly described by its inertial effect, but also by its aerodynamic effect (Jusufi et al. 2011) The aerodynamic effect in this case consists in the amount of air friction applied on the animal during the aerial movement, which varies according to its speed and surface properties. Righting of species with higher body mass was explained by inertial effects, for example, cats (Kane and Scher 1969) or humans (Passerello and Huston 1971). In contrast, righting of smaller mass species was explained by aerodynamic effect, for example, hoverflies (Verbe et al. 2020), dragonflies (Fabian et al. 2021), or stick insects (Zeng et al. 2017). It is also known that gliding animals with patagia, such as flying squirrels (Bishop 2006), sugar gliders (Bishop 2007), flying lizards (McGuire and Dudley 2011), or flying fish (Davenport 1994) can control their body orientation during gliding using their patagia’s large surface area of skin.

Meanwhile, animals lacking patagia can nonetheless use aerodynamic effects in addition to the inertial effects to steer their body (Patel et al. 2016; Yeaton et al. 2020; Siddall et al. 2021). They can re-orientate their bodies using aerodynamically stable postures via active modulation of their appendages (Ribak et al. 2013; Zeng et al. 2017).

In our squirrel case, although its tail mass is usually no greater than 3% of the total body mass, the tail is nevertheless featured with a quite bushy fur (a high volume of hair covering the tail) and projected size of area of tails can reach around 55% of the body size in a side view during jumping (measured from our videos capturing *Sciurus carolinensis* jumping). Interestingly, the bushyness of the tail can greatly differ among species, and may induce a non-negligible aerodynamic effect. But this parameter is rarely taken into account in the locomotor analyses. In the robotic field, it has been also shown that aerodynamic force enables a 45 g robot to steer with a rotational sail (5% of the body mass) (Kohut et al. 2013).

Body righting torque from the tail inertial effect $\tau_{I}$ is represented as below using the model in the “Model analysis” section

(12) $$\tau_{I}=I\prime_{\text{tl}}\dot{\omega }$$

(13) $$=\frac{m_{t}}{4}\left(\frac{4l_{\text{tl}}^{2}}{3}+\frac{l_{\text{tw}}^{2}}{4}\right)\dot{\omega }$$

(14) $$\propto m_{t}l_{\text{tl}}^{2}\dot{\omega }.$$

Here, $I\prime_{\text{tl}},\;m_{t},\;l_{\text{tl}},\;l_{\text{tw}}$, and $\dot{\omega }$ stand for the tail moment from tail spinning point, the tail mass, the tail length, the tail width, and the tail spinning angular acceleration. This torque is proportional to tail mass, square of tail length, and tail angular acceleration. If we use the values from Table 1 and take the time to accelerate the tail to its maximum rate (500 rpm) as 29 ms (i.e., the duration of phase 1 in launch 1; Fig. 1), we can estimate the inertial torque acting on the body as the tail accelerates as 38 mNm.

As the tail accelerates, it will also generate an aerodynamic reaction force. The aerodynamic torque from tail rotation can be calculated using a blade element method, integrating aerodynamic forces along the tail length (neglecting any induced air velocity):

(15) $$\Delta \tau_{A}=\frac{1}{2}\rho v^{2}C_{D}\Delta S\cdot x\;\text{cos}\;\theta_{\text{bend}},$$

where $\Delta \tau_{A}$ represents drag torque from an infinitesimal part *x* m distant from the tail spinning point and *ρ*, $C_{D},\;\Delta S$, and *v* stand for density of the air, drag coefficient, size of infinitesimal area, and speed of the area. $\theta_{\text{bend}}$ is the bend angle of the tail with respect to the body. By treating tail width as constant, this expression can be integrated along the tail length to give torque

(16) $$\tau_{A}=\frac{1}{2}\rho C_{D}\;\text{cos}\;\theta_{\text{bend}}\int_{0}^{l_{tl}}l_{\text{tw}}{\left(x\omega \right)}^{2}xdx$$

(17) $$=\frac{1}{8}\rho C_{D}l_{\text{tl}}^{4}l_{\text{tw}}\omega^{2}\;\text{cos}\;\theta_{\text{bend}}$$

(18) $$\propto l_{\text{tl}}^{4}l_{\text{tw}}\omega^{2}.$$

The tail Reynolds number of the squirrel is $1\times 10^{4}$, based on speed at 75% of tail length. Owing to the high angular rate of the squirrel’s tail, this Reynolds number is comparable to the flow about the tail of a rapidly moving cheetah as measured in Patel et al. (2016), for which the drag coefficient of a “bushy” cheetah tail was measured as 1.1, and the effective width (i.e., the width of an equivalent rigid cylinder) is 59% of the tail’s width including hair length. We can estimate the tail width (including hair) of the squirrel to be equivalent to the narrowest body measurement (48 mm). Using 59% effective width (28 mm), a *C*_D_ of 1.1, and setting *θ*_bend_ = 90°, we can calculate the torque produced by the tail as 6.9 mNm, equivalent to a force acting at the tail midspan of 66 mN (7 g or 60% tail weight, for context).

The aerodynamic torque is smaller but comparable then to the inertial torque (*τ*_I_ = 5.5*τ*_A_), and can be sustained for longer periods of time, unlike the inertial reaction which requires continuous acceleration and so is necessarily limited in duration. From this aspect, the light, long, and wide bushy tails make sense—squirrel tails significantly increase their aerodynamic area with their fur. Though it should be noted that the inertial reaction is larger, and has an instantaneous response, whereas aerodynamic reactions require time for the appendage to reach velocity.

This probable aerodynamic effect, associated with our model-based analysis showing that slight anatomical differences have large effects on the effectiveness of self-righting performance, demonstrates that the specific shape of squirrels’ tail is probably the result of a functional adaptation. Indeed, arboreal specialists such as squirrels, which frequently engage in acrobatic behaviors, are subjected to the need for successful displacement ability within their complex environment. Since falls from the canopy are potentially lethal, the tail (due to its function for self-righting and maintaining orientation in the air) can be expected to be subjected to substantial selective pressures, as well as other mechanisms such as grasping or jumping ability. In this respect, it is very probable that the long and bushy tail of arboreal mammals might help improving their efficient locomotor maneuverability.

## Conclusion

In this study, we combined behavioral analysis, modelization, and robotic implementation to investigate the righting mechanism following a fall in the eastern gray squirrel. Our results suggest that the righting pattern can be reasonably described by three phases, in which the body and tail move in coordinated synergy to stabilize the body trajectory for successful righting and landing. We also found that the specific tail dimension of squirrel has a substantial impact in its inertial effect, and that slight modifications of morphology can dramatically impact the outcome of the righting success. Moreover, our specifically designed robot allowed us to implement our optimized tail trajectories directly onto physical hardware and demonstrate the aerial righting. Further development of the biorobotic physical model can investigate the tail’s role in other examples of arboreal acrobatics, including characterizing fluid dynamic effects (e.g., Hsieh and Lauder 2004; Wolf et al. 2020; Shield et al. 2021). As well as offering insight into animal locomotion, enhanced arboreal abilities could open new applications in arboreal robotics.

Finally, we propose that other parameters, such as the bushyness of the tail, caused by the volume of fur, might also has an important role in the righting mechanism by inducing a significant aerodynamic effect. To offer further perspectives, we should explore other arboreal models, with varying body masses, tail shapes, and locomotor adaptations (e.g., Hayati et al. [2017], carnivorans or primates), in order to improve our knowledge on the relationship between tail morphology and functional adaptation.

## Data availability

The simulation model is available on an online repository: https://github.com/tsfk9981/squirrel_for_public.

## Supplementary Material

icab023_Supplementary_Data

## Notes

From the symposium “An evolutionary tail: Evo-Devo, structure, and function of post-anal appendages” presented at the virtual annual meeting of the Society for Integrative and Comparative Biology, January 3 - February 28, 2021
